# Supplementary material for: Mapping the evolution of fertility support policies in China: A content and instrumental analysis
Source: PLoS One. 2025 Oct 9;20(10):e0332137. doi: 10.1371/journal.pone.0332137 (PMC12510515; doi:10.1371/journal.pone.0332137)
Supplement: S1 Appendix — (ZIP) [file pone.0332137.s001.zip › S1 Appendix. 226 original policy documents/135-中共中央、国务院关于落实发展新理念加快农业现代化实现全面小康目标的若干意见(FBM-CLI-16-263507).doc]

中共中央、国务院关于落实发展新理念加快农业现代化实现全面小康目标的若干意见

**发布部门：** [中国共产党中央委员会](https://www.pkulaw.com/law/chl?Aggs.IssueDepartment=a01) [国务院](https://www.pkulaw.com/law/chl?Aggs.IssueDepartment=5)

**发文字号：**中发〔2016〕1号

**发布日期：**2015.12.31

**实施日期：**2015.12.31

**时效性：** [现行有效](https://www.pkulaw.com/law/chl?Aggs.TimelinessDic=01)

**效力级别：** [党内法规制度](https://www.pkulaw.com/law/chl?Aggs.EffectivenessDic=XR12)

**法规类别：** [农业管理](https://www.pkulaw.com/law/chl?Aggs.Category=06101)

**专题分类：** [中央一号文件](https://www.pkulaw.com/law/chl?Aggs.SpecialType=027)

中共中央、国务院关于落实发展新理念加快农业现代化实现全面小康目标的若干意见
（2015年12月31日 中发[2016]1号）

　　党的十八届五中全会通过的《[中共中央关于制定国民经济和社会发展第十三个五年规划的建议](https://www.pkulaw.com/chl/e69521f41400e9d7bdfb.html)》，对做好新时期农业农村工作作出了重要部署。各地区各部门要牢固树立和深入贯彻落实创新、协调、绿色、开放、共享的发展理念，大力推进农业现代化，确保亿万农民与全国人民一道迈入全面小康社会。
　　“十二五”时期，是农业农村发展的又一个黄金期。粮食连年高位增产，实现了农业综合生产能力质的飞跃；农民收入持续较快增长，扭转了城乡居民收入差距扩大的态势；农村基础设施和公共服务明显改善，提高了农民群众的民生保障水平；农村社会和谐稳定，夯实了党在农村的执政基础。实践证明，党的“三农”政策是完全正确的，亿万农民是衷心拥护的。
　　当前，我国农业农村发展环境发生重大变化，既面临诸多有利条件，又必须加快破解各种难题。一方面，加快补齐农业农村短板成为全党共识，为开创“三农”工作新局面汇聚强大推动力；新型城镇化加快推进，为以工促农、以城带乡带来持续牵引力；城乡居民消费结构加快升级，为拓展农业农村发展空间增添巨大带动力；新一轮科技革命和产业变革正在孕育兴起，为农业转型升级注入强劲驱动力；农村各项改革全面展开，为农业农村现代化提供不竭源动力。另一方面，在经济发展新常态背景下，如何促进农民收入稳定较快增长，加快缩小城乡差距，确保如期实现全面小康，是必须完成的历史任务；在资源环境约束趋紧背景下，如何加快转变农业发展方式，确保粮食等重要农产品有效供给，实现绿色发展和资源永续利用，是必须破解的现实难题；在受国际农产品市场影响加深背景下，如何统筹利用国际国内两个市场、两种资源，提升我国农业竞争力，赢得参与国际市场竞争的主动权，是必须应对的重大挑战。农业是全面建成小康社会、实现现代化的基础。我们一定要切实增强做好“三农”工作的责任感、使命感、紧迫感，任何时候都不能忽视农业、忘记农民、淡漠农村，在认识的高度、重视的程度、投入的力度上保持好势头，始终把解决好“三农”问题作为全党工作重中之重，坚持强农惠农富农政策不减弱，推进农村全面小康建设不松劲，加快发展现代农业，加快促进农民增收，加快建设社会主义新农村，不断巩固和发展农业农村好形势。
　　“十三五”时期推进农村改革发展，要高举中国特色社会主义伟大旗帜，全面贯彻党的十八大和十八届三中、四中、五中全会精神，以邓小平理论、“三个代表”重要思想、科学发展观为指导，深入贯彻习近平总书记系列重要讲话精神，坚持全面建成小康社会、全面深化改革、全面依法治国、全面从严治党的战略布局，把坚持农民主体地位、增进农民福祉作为农村一切工作的出发点和落脚点，用发展新理念破解“三农”新难题，厚植农业农村发展优势，加大创新驱动力度，推进农业供给侧结构性改革，加快转变农业发展方式，保持农业稳定发展和农民持续增收，走产出高效、产品安全、资源节约、环境友好的农业现代化道路，推动新型城镇化与新农村建设双轮驱动、互促共进，让广大农民平等参与现代化进程、共同分享现代化成果。
　　到2020年，现代农业建设取得明显进展，粮食产能进一步巩固提升，国家粮食安全和重要农产品供给得到有效保障，农产品供给体系的质量和效率显著提高；农民生活达到全面小康水平，农村居民人均收入比2010年翻一番，城乡居民收入差距继续缩小；我国现行标准下农村贫困人口实现脱贫，贫困县全部摘帽，解决区域性整体贫困；农民素质和农村社会文明程度显著提升，社会主义新农村建设水平进一步提高；农村基本经济制度、农业支持保护制度、农村社会治理制度、城乡发展一体化体制机制进一步完善。
　　一、持续夯实现代农业基础，提高农业质量效益和竞争力
　　大力推进农业现代化，必须着力强化物质装备和技术支撑，着力构建现代农业产业体系、生产体系、经营体系，实施藏粮于地、藏粮于技战略，推动粮经饲统筹、农林牧渔结合、种养加一体、一二三产业融合发展，让农业成为充满希望的朝阳产业。
　　1．大规模推进高标准农田建设。加大投入力度，整合建设资金，创新投融资机制，加快建设步伐，到2020年确保建成8亿亩、力争建成10亿亩集中连片、旱涝保收、稳产高产、生态友好的高标准农田。整合完善建设规划，统一建设标准、统一监管考核、统一上图入库。提高建设标准，充实建设内容，完善配套设施。优化建设布局，优先在粮食主产区建设确保口粮安全的高标准农田。健全管护监督机制，明确管护责任主体。将高标准农田划为永久基本农田，实行特殊保护。将高标准农田建设情况纳入地方各级政府耕地保护责任目标考核内容。
　　2．大规模推进农田水利建设。把农田水利作为农业基础设施建设的重点，到2020年农田有效灌溉面积达到10亿亩以上，农田灌溉水有效利用系数提高到0.55以上。加快重大水利工程建设。积极推进江河湖库水系连通工程建设，优化水资源空间格局，增加水环境容量。加快大中型灌区建设及续建配套与节水改造、大型灌排泵站更新改造。完善小型农田水利设施，加强农村河塘清淤整治、山丘区“五小水利”、田间渠系配套、雨水集蓄利用、牧区节水灌溉饲草料地建设。大力开展区域规模化高效节水灌溉行动，积极推广先进适用节水灌溉技术。继续实施中小河流治理和山洪、地质灾害防治。扩大开发性金融支持水利工程建设的规模和范围。稳步推进农业水价综合改革，实行农业用水总量控制和定额管理，合理确定农业水价，建立节水奖励和精准补贴机制，提高农业用水效率。完善用水权初始分配制度，培育水权交易市场。深化小型农田水利工程产权制度改革，创新运行管护机制。鼓励社会资本参与小型农田水利工程建设与管护。
　　3．强化现代农业科技创新推广体系建设。农业科技创新能力总体上达到发展中国家领先水平，力争在农业重大基础理论、前沿核心技术方面取得一批达到世界先进水平的成果。统筹协调各类农业科技资源，建设现代农业产业科技创新中心，实施农业科技创新重点专项和工程，重点突破生物育种、农机装备、智能农业、生态环保等领域关键技术。强化现代农业产业技术体系建设。加强农业转基因技术研发和监管，在确保安全的基础上慎重推广。加快研发高端农机装备及关键核心零部件，提升主要农作物生产全程机械化水平，推进林业装备现代化。大力推进“互联网＋”现代农业，应用物联网、云计算、大数据、移动互联等现代信息技术，推动农业全产业链改造升级。大力发展智慧气象和农业遥感技术应用。深化农业科技体制改革，完善成果转化激励机制，制定促进协同创新的人才流动政策。加强农业知识产权保护，严厉打击侵权行为。深入开展粮食绿色高产高效创建。健全适应现代农业发展要求的农业科技推广体系，对基层农技推广公益性与经营性服务机构提供精准支持，引导高等学校、科研院所开展农技服务。推行科技特派员制度，鼓励支持科技特派员深入一线创新创业。发挥农村专业技术协会的作用。鼓励发展农业高新技术企业。深化国家现代农业示范区、国家农业科技园区建设。
　　4．加快推进现代种业发展。大力推进育繁推一体化，提升种业自主创新能力，保障国家种业安全。深入推进种业领域科研成果权益分配改革，探索成果权益分享、转移转化和科研人员分类管理机制。实施现代种业建设工程和种业自主创新重大工程。全面推进良种重大科研联合攻关，培育和推广适应机械化生产、优质高产多抗广适新品种，加快主要粮食作物新一轮品种更新换代。加快推进海南、甘肃、四川国家级育种制种基地和区域性良种繁育基地建设。强化企业育种创新主体地位，加快培育具有国际竞争力的现代种业企业。实施畜禽遗传改良计划，加快培育优异畜禽新品种。开展种质资源普查，加大保护利用力度。贯彻落实[种子法](https://www.pkulaw.com/chl/497e015a9d1f0289bdfb.html)，全面推进依法治种。加大种子打假护权力度。
　　5．发挥多种形式农业适度规模经营引领作用。坚持以农户家庭经营为基础，支持新型农业经营主体和新型农业服务主体成为建设现代农业的骨干力量，充分发挥多种形式适度规模经营在农业机械和科技成果应用、绿色发展、市场开拓等方面的引领功能。完善财税、信贷保险、用地用电、项目支持等政策，加快形成培育新型农业经营主体的政策体系，进一步发挥财政资金引导作用，撬动规模化经营主体增加生产性投入。适应新型农业经营主体和服务主体发展需要，允许将集中连片整治后新增加的部分耕地，按规定用于完善农田配套设施。探索开展粮食生产规模经营主体营销贷款改革试点。积极培育家庭农场、专业大户、农民合作社、农业产业化龙头企业等新型农业经营主体。支持多种类型的新型农业服务主体开展代耕代种、联耕联种、土地托管等专业化规模化服务。加强气象为农服务体系建设。实施农业社会化服务支撑工程，扩大政府购买农业公益性服务机制创新试点。加快发展农业生产性服务业。完善工商资本租赁农地准入、监管和风险防范机制。健全县乡农村经营管理体系，加强对土地流转和规模经营的管理服务。
　　6．加快培育新型职业农民。将职业农民培育纳入国家教育培训发展规划，基本形成职业农民教育培训体系，把职业农民培养成建设现代农业的主导力量。办好农业职业教育，将全日制农业中等职业教育纳入国家资助政策范围。依托高等教育、中等职业教育资源，鼓励农民通过“半农半读”等方式就地就近接受职业教育。开展新型农业经营主体带头人培育行动，通过5年努力使他们基本得到培训。加强涉农专业全日制学历教育，支持农业院校办好涉农专业，健全农业广播电视学校体系，定向培养职业农民。引导有志投身现代农业建设的农村青年、返乡农民工、农技推广人员、农村大中专毕业生和退役军人等加入职业农民队伍。优化财政支农资金使用，把一部分资金用于培养职业农民。总结各地经验，建立健全职业农民扶持制度，相关政策向符合条件的职业农民倾斜。鼓励有条件的地方探索职业农民养老保险办法。
　　7．优化农业生产结构和区域布局。树立大食物观，面向整个国土资源，全方位、多途径开发食物资源，满足日益多元化的食物消费需求。在确保谷物基本自给、口粮绝对安全的前提下，基本形成与市场需求相适应、与资源禀赋相匹配的现代农业生产结构和区域布局，提高农业综合效益。启动实施种植业结构调整规划，稳定水稻和小麦生产，适当调减非优势区玉米种植。支持粮食主产区建设粮食生产核心区。扩大粮改饲试点，加快建设现代饲草料产业体系。合理调整粮食统计口径。制定划定粮食生产功能区和大豆、棉花、油料、糖料蔗等重要农产品生产保护区的指导意见。积极推进马铃薯主食开发。加快现代畜牧业建设，根据环境容量调整区域养殖布局，优化畜禽养殖结构，发展草食畜牧业，形成规模化生产、集约化经营为主导的产业发展格局。启动实施种养结合循环农业示范工程，推动种养结合、农牧循环发展。加强渔政渔港建设。大力发展旱作农业、热作农业、优质特色杂粮、特色经济林、木本油料、竹藤花卉、林下经济。
　　8．统筹用好国际国内两个市场、两种资源。完善农业对外开放战略布局，统筹农产品进出口，加快形成农业对外贸易与国内农业发展相互促进的政策体系，实现补充国内市场需求、促进结构调整、保护国内产业和农民利益的有机统一。加大对农产品出口支持力度，巩固农产品出口传统优势，培育新的竞争优势，扩大特色和高附加值农产品出口。确保口粮绝对安全，利用国际资源和市场，优化国内农业结构，缓解资源环境压力。优化重要农产品进口的全球布局，推进进口来源多元化，加快形成互利共赢的稳定经贸关系。健全贸易救济和产业损害补偿机制。强化边境管理，深入开展综合治理，打击农产品走私。统筹制定和实施农业对外合作规划。加强与“一带一路”沿线国家和地区及周边国家和地区的农业投资、贸易、科技、动植物检疫合作。支持我国企业开展多种形式的跨国经营，加强农产品加工、储运、贸易等环节合作，培育具有国际竞争力的粮商和农业企业集团。

　　二、加强资源保护和生态修复，推动农业绿色发展
　　推动农业可持续发展，必须确立发展绿色农业就是保护生态的观念，加快形成资源利用高效、生态系统稳定、产地环境良好、产品质量安全的农业发展新格局。
　　9．加强农业资源保护和高效利用。基本建立农业资源有效保护、高效利用的政策和技术支撑体系，从根本上改变开发强度过大、利用方式粗放的状况。坚持最严格的耕地保护制度，坚守耕地红线，全面划定永久基本农田，大力实施农村土地整治，推进耕地数量、质量、生态“三位一体”保护。落实和完善耕地占补平衡制度，坚决防止占多补少、占优补劣、占水田补旱地，严禁毁林开垦。全面推进建设占用耕地耕作层剥离再利用。实行建设用地总量和强度双控行动，严格控制农村集体建设用地规模。完善耕地保护补偿机制。实施耕地质量保护与提升行动，加强耕地质量调查评价与监测，扩大东北黑土地保护利用试点规模。实施渤海粮仓科技示范工程，加大科技支撑力度，加快改造盐碱地。创建农业可持续发展试验示范区。划定农业空间和生态空间保护红线。落实最严格的水资源管理制度，强化水资源管理“三条红线”刚性约束，实行水资源消耗总量和强度双控行动。加强地下水监测，开展超采区综合治理。落实河湖水域岸线用途管制制度。加强自然保护区建设与管理，对重要生态系统和物种资源实行强制性保护。实施濒危野生动植物抢救性保护工程，建设救护繁育中心和基因库。强化野生动植物进出口管理，严厉打击象牙等濒危野生动植物及其制品非法交易。
　　10．加快农业环境突出问题治理。基本形成改善农业环境的政策法规制度和技术路径，确保农业生态环境恶化趋势总体得到遏制，治理明显见到成效。实施并完善农业环境突出问题治理总体规划。加大农业面源污染防治力度，实施化肥农药零增长行动，实施种养业废弃物资源化利用、无害化处理区域示范工程。积极推广高效生态循环农业模式。探索实行耕地轮作休耕制度试点，通过轮作、休耕、退耕、替代种植等多种方式，对地下水漏斗区、重金属污染区、生态严重退化地区开展综合治理。实施全国水土保持规划。推进荒漠化、石漠化、水土流失综合治理。
　　11．加强农业生态保护和修复。实施山水林田湖生态保护和修复工程，进行整体保护、系统修复、综合治理。到2020年森林覆盖率提高到23%以上，湿地面积不低于8亿亩。扩大新一轮退耕还林还草规模。扩大退牧还草工程实施范围。实施新一轮草原生态保护补助奖励政策，适当提高补奖标准。实施湿地保护与恢复工程，开展退耕还湿。建立沙化土地封禁保护制度。加强历史遗留工矿废弃和自然灾害损毁土地复垦利用。开展大规模国土绿化行动，增加森林面积和蓄积量。加强三北、长江、珠江、沿海防护林体系等林业重点工程建设。继续推进京津风沙源治理。完善天然林保护制度，全面停止天然林商业性采伐。完善海洋渔业资源总量管理制度，严格实行休渔禁渔制度，开展近海捕捞限额管理试点，按规划实行退养还滩。加快推进水生态修复工程建设。建立健全生态保护补偿机制，开展跨地区跨流域生态保护补偿试点。编制实施耕地、草原、河湖休养生息规划。
　　12．实施食品安全战略。加快完善食品安全国家标准，到2020年农兽药残留限量指标基本与国际食品法典标准接轨。加强产地环境保护和源头治理，实行严格的农业投入品使用管理制度。推广高效低毒低残留农药，实施兽用抗菌药治理行动。创建优质农产品和食品品牌。继续推进农业标准化示范区、园艺作物标准园、标准化规模养殖场（小区）、水产健康养殖场建设。实施动植物保护能力提升工程。加快健全从农田到餐桌的农产品质量和食品安全监管体系，建立全程可追溯、互联共享的信息平台，加强标准体系建设，健全风险监测评估和检验检测体系。落实生产经营主体责任，严惩各类食品安全违法犯罪。实施食品安全创新工程。加强基层监管机构能力建设，培育职业化检查员，扩大抽检覆盖面，加强日常检查。加快推进病死畜禽无害化处理与养殖业保险联动机制建设。规范畜禽屠宰管理，加强人畜共患传染病防治。强化动植物疫情疫病监测防控和边境、口岸及主要物流通道检验检疫能力建设，严防外来有害物种入侵。深入开展食品安全城市和农产品质量安全县创建，开展农村食品安全治理行动。强化食品安全责任制，把保障农产品质量和食品安全作为衡量党政领导班子政绩的重要考核指标。

　　三、推进农村产业融合，促进农民收入持续较快增长
　　大力推进农民奔小康，必须充分发挥农村的独特优势，深度挖掘农业的多种功能，培育壮大农村新产业新业态，推动产业融合发展成为农民增收的重要支撑，让农村成为可以大有作为的广阔天地。
　　13．推动农产品加工业转型升级。加强农产品加工技术创新，促进农产品初加工、精深加工及综合利用加工协调发展，提高农产品加工转化率和附加值，增强对农民增收的带动能力。加强规划和政策引导，促进主产区农产品加工业加快发展，支持粮食主产区发展粮食深加工，形成一批优势产业集群。开发拥有自主知识产权的技术装备，支持农产品加工设备改造提升，建设农产品加工技术集成基地。培育一批农产品精深加工领军企业和国内外知名品牌。强化环保、能耗、质量、安全等标准作用，促进农产品加工企业优胜劣汰。完善农产品产地初加工补助政策。研究制定促进农产品加工业发展的意见。
　　14．加强农产品流通设施和市场建设。健全统一开放、布局合理、竞争有序的现代农产品市场体系，在搞活流通中促进农民增收。加快农产品批发市场升级改造，完善流通骨干网络，加强粮食等重要农产品仓储物流设施建设。完善跨区域农产品冷链物流体系，开展冷链标准化示范，实施特色农产品产区预冷工程。推动公益性农产品市场建设。支持农产品营销公共服务平台建设。开展降低农产品物流成本行动。促进农村电子商务加快发展，形成线上线下融合、农产品进城与农资和消费品下乡双向流通格局。加快实现行政村宽带全覆盖，创新电信普遍服务补偿机制，推进农村互联网提速降费。加强商贸流通、供销、邮政等系统物流服务网络和设施建设与衔接，加快完善县乡村物流体系。实施“快递下乡”工程。鼓励大型电商平台企业开展农村电商服务，支持地方和行业健全农村电商服务体系。建立健全适应农村电商发展的农产品质量分级、采后处理、包装配送等标准体系。深入开展电子商务进农村综合示范。加大信息进村入户试点力度。
　　15．大力发展休闲农业和乡村旅游。依托农村绿水青山、田园风光、乡土文化等资源，大力发展休闲度假、旅游观光、养生养老、创意农业、农耕体验、乡村手工艺等，使之成为繁荣农村、富裕农民的新兴支柱产业。强化规划引导，采取以奖代补、先建后补、财政贴息、设立产业投资基金等方式扶持休闲农业与乡村旅游业发展，着力改善休闲旅游重点村进村道路、宽带、停车场、厕所、垃圾污水处理等基础服务设施。积极扶持农民发展休闲旅游业合作社。引导和支持社会资本开发农民参与度高、受益面广的休闲旅游项目。加强乡村生态环境和文化遗存保护，发展具有历史记忆、地域特点、民族风情的特色小镇，建设一村一品、一村一景、一村一韵的魅力村庄和宜游宜养的森林景区。依据各地具体条件，有规划地开发休闲农庄、乡村酒店、特色民宿、自驾露营、户外运动等乡村休闲度假产品。实施休闲农业和乡村旅游提升工程、振兴中国传统手工艺计划。开展农业文化遗产普查与保护。支持有条件的地方通过盘活农村闲置房屋、集体建设用地、“四荒地”、可用林场和水面等资产资源发展休闲农业和乡村旅游。将休闲农业和乡村旅游项目建设用地纳入土地利用总体规划和年度计划合理安排。
　　16．完善农业产业链与农民的利益联结机制。促进农业产加销紧密衔接、农村一二三产业深度融合，推进农业产业链整合和价值链提升，让农民共享产业融合发展的增值收益，培育农民增收新模式。支持供销合作社创办领办农民合作社，引领农民参与农村产业融合发展、分享产业链收益。创新发展订单农业，支持农业产业化龙头企业建设稳定的原料生产基地、为农户提供贷款担保和资助订单农户参加农业保险。鼓励发展股份合作，引导农户自愿以土地经营权等入股龙头企业和农民合作社，采取“保底收益＋按股分红”等方式，让农户分享加工销售环节收益，建立健全风险防范机制。加强农民合作社示范社建设，支持合作社发展农产品加工流通和直供直销。通过政府与社会资本合作、贴息、设立基金等方式，带动社会资本投向农村新产业新业态。实施农村产业融合发展试点示范工程。财政支农资金使用要与建立农民分享产业链利益机制相联系。巩固和完善“合同帮农”机制，为农民和涉农企业提供法律咨询、合同示范文本、纠纷调处等服务。

　　四、推动城乡协调发展，提高新农村建设水平
　　加快补齐农业农村短板，必须坚持工业反哺农业、城市支持农村，促进城乡公共资源均衡配置、城乡要素平等交换，稳步提高城乡基本公共服务均等化水平。
　　17．加快农村基础设施建设。把国家财政支持的基础设施建设重点放在农村，建好、管好、护好、运营好农村基础设施，实现城乡差距显著缩小。健全农村基础设施投入长效机制，促进城乡基础设施互联互通、共建共享。强化农村饮用水水源保护。实施农村饮水安全巩固提升工程。推动城镇供水设施向周边农村延伸。加快实施农村电网改造升级工程，开展农村“低电压”综合治理，发展绿色小水电。加快实现所有具备条件的乡镇和建制村通硬化路、通班车，推动一定人口规模的自然村通公路。创造条件推进城乡客运一体化。加快国有林区防火应急道路建设。将农村公路养护资金逐步纳入地方财政预算。发展农村规模化沼气。加大农村危房改造力度，统筹搞好农房抗震改造，通过贷款贴息、集中建设公租房等方式，加快解决农村困难家庭的住房安全问题。加强农村防灾减灾体系建设。研究出台创新农村基础设施投融资体制机制的政策意见。
　　18．提高农村公共服务水平。把社会事业发展的重点放在农村和接纳农业转移人口较多的城镇，加快推动城镇公共服务向农村延伸。加快发展农村学前教育，坚持公办民办并举，扩大农村普惠性学前教育资源。建立城乡统一、重在农村的义务教育经费保障机制。全面改善贫困地区义务教育薄弱学校基本办学条件，改善农村学校寄宿条件，办好乡村小规模学校，推进学校标准化建设。加快普及高中阶段教育，逐步分类推进中等职业教育免除学杂费，率先从建档立卡的家庭经济困难学生实施普通高中免除学杂费，实现家庭经济困难学生资助全覆盖。深入实施农村贫困地区定向招生等专项计划，对民族自治县实现全覆盖。加强乡村教师队伍建设，拓展教师补充渠道，推动城镇优秀教师向乡村学校流动。办好农村特殊教育。整合城乡居民基本医疗保险制度，适当提高政府补助标准、个人缴费和受益水平。全面实施城乡居民大病保险制度。健全城乡医疗救助制度。完善城乡居民养老保险参保缴费激励约束机制，引导参保人员选择较高档次缴费。改进农村低保申请家庭经济状况核查机制，实现农村低保制度与扶贫开发政策有效衔接。建立健全农村留守儿童和妇女、老人关爱服务体系。建立健全农村困境儿童福利保障和未成年人社会保护制度。积极发展农村社会工作和志愿服务。切实维护农村妇女在财产分配、婚姻生育、政治参与等方面的合法权益，让女性获得公平的教育机会、就业机会、财产性收入、金融资源。加强农村养老服务体系、残疾人康复和供养托养设施建设。深化农村殡葬改革，依法管理、改进服务。推进农村基层综合公共服务资源优化整合。全面加强农村公共文化服务体系建设，继续实施文化惠民项目。在农村建设基层综合性文化服务中心，整合基层宣传文化、党员教育、科学普及、体育健身等设施，整合文化信息资源共享、农村电影放映、农家书屋等项目，发挥基层文化公共设施整体效应。
　　19．开展农村人居环境整治行动和美丽宜居乡村建设。遵循乡村自身发展规律，体现农村特点，注重乡土味道，保留乡村风貌，努力建设农民幸福家园。科学编制县域乡村建设规划和村庄规划，提升民居设计水平，强化乡村建设规划许可管理。继续推进农村环境综合整治，完善以奖促治政策，扩大连片整治范围。实施农村生活垃圾治理5年专项行动。采取城镇管网延伸、集中处理和分散处理等多种方式，加快农村生活污水治理和改厕。全面启动村庄绿化工程，开展生态乡村建设，推广绿色建材，建设节能农房。开展农村宜居水环境建设，实施农村清洁河道行动，建设生态清洁型小流域。发挥好村级公益事业一事一议财政奖补资金作用，支持改善村内公共设施和人居环境。普遍建立村庄保洁制度。坚持城乡环境治理并重，逐步把农村环境整治支出纳入地方财政预算，中央财政给予差异化奖补，政策性金融机构提供长期低息贷款，探索政府购买服务、专业公司一体化建设运营机制。加大传统村落、民居和历史文化名村名镇保护力度。开展生态文明示范村镇建设。鼓励各地因地制宜探索各具特色的美丽宜居乡村建设模式。
　　20．推进农村劳动力转移就业创业和农民工市民化。健全农村劳动力转移就业服务体系，大力促进就地就近转移就业创业，稳定并扩大外出农民工规模，支持农民工返乡创业。大力发展特色县域经济和农村服务业，加快培育中小城市和特色小城镇，增强吸纳农业转移人口能力。加大对农村灵活就业、新就业形态的支持。鼓励各地设立农村妇女就业创业基金，加大妇女小额担保贷款实施力度，加强妇女技能培训，支持农村妇女发展家庭手工业。实施新生代农民工职业技能提升计划，开展农村贫困家庭子女、未升学初高中毕业生、农民工、退役军人免费接受职业培训行动。依法维护农民工合法劳动权益，完善城乡劳动者平等就业制度，建立健全农民工工资支付保障长效机制。进一步推进户籍制度改革，落实1亿左右农民工和其他常住人口在城镇定居落户的目标，保障进城落户农民工与城镇居民有同等权利和义务，加快提高户籍人口城镇化率。全面实施居住证制度，建立健全与居住年限等条件相挂钩的基本公共服务提供机制，努力实现基本公共服务常住人口全覆盖。落实和完善农民工随迁子女在当地参加中考、高考政策。将符合条件的农民工纳入城镇社会保障和城镇住房保障实施范围。健全财政转移支付同农业转移人口市民化挂钩机制，建立城镇建设用地增加规模同吸纳农业转移人口落户数量挂钩机制。维护进城落户农民土地承包权、宅基地使用权、集体收益分配权，支持引导其依法自愿有偿转让上述权益。
　　21．实施脱贫攻坚工程。实施精准扶贫、精准脱贫，因人因地施策，分类扶持贫困家庭，坚决打赢脱贫攻坚战。通过产业扶持、转移就业、易地搬迁等措施解决5000万左右贫困人口脱贫；对完全或部分丧失劳动能力的2000多万贫困人口，全部纳入低保覆盖范围，实行社保政策兜底脱贫。实行脱贫工作责任制，进一步完善中央统筹、省（自治区、直辖市）负总责、市（地）县抓落实的工作机制。各级党委和政府要把脱贫攻坚作为重大政治任务扛在肩上，各部门要步调一致、协同作战、履职尽责，切实把民生项目、惠民政策最大限度向贫困地区倾斜。广泛动员社会各方面力量积极参与扶贫开发。实行最严格的脱贫攻坚考核督查问责。

　　五、深入推进农村改革，增强农村发展内生动力
　　破解“三农”难题，必须坚持不懈推进体制机制创新，着力破除城乡二元结构的体制障碍，激发亿万农民创新创业活力，释放农业农村发展新动能。
　　22．改革完善粮食等重要农产品价格形成机制和收储制度。坚持市场化改革取向与保护农民利益并重，采取“分品种施策、渐进式推进”的办法，完善农产品市场调控制度。继续执行并完善稻谷、小麦最低收购价政策。深入推进新疆棉花、东北地区大豆目标价格改革试点。按照市场定价、价补分离的原则，积极稳妥推进玉米收储制度改革，在使玉米价格反映市场供求关系的同时，综合考虑农民合理收益、财政承受能力、产业链协调发展等因素，建立玉米生产者补贴制度。按照政策性职能和经营性职能分离的原则，改革完善中央储备粮管理体制。深化国有粮食企业改革，发展多元化市场购销主体。科学确定粮食等重要农产品国家储备规模，完善吞吐调节机制。
　　23．健全农业农村投入持续增长机制。优先保障财政对农业农村的投入，坚持将农业农村作为国家固定资产投资的重点领域，确保力度不减弱、总量有增加。充分发挥财政政策导向功能和财政资金杠杆作用，鼓励和引导金融资本、工商资本更多投向农业农村。加大专项建设基金对扶贫、水利、农村产业融合、农产品批发市场等“三农”领域重点项目和工程支持力度。发挥规划引领作用，完善资金使用和项目管理办法，多层级深入推进涉农资金整合统筹，实施省级涉农资金管理改革和市县涉农资金整合试点，改进资金使用绩效考核办法。将种粮农民直接补贴、良种补贴、农资综合补贴合并为农业支持保护补贴，重点支持耕地地力保护和粮食产能提升。完善农机购置补贴政策。用3年左右时间建立健全全国农业信贷担保体系，2016年推动省级农业信贷担保机构正式建立并开始运营。加大对农产品主产区和重点生态功能区的转移支付力度。完善主产区利益补偿机制。逐步将农垦系统纳入国家农业支持和民生改善政策覆盖范围。研究出台完善农民收入增长支持政策体系的指导意见。
　　24．推动金融资源更多向农村倾斜。加快构建多层次、广覆盖、可持续的农村金融服务体系，发展农村普惠金融，降低融资成本，全面激活农村金融服务链条。进一步改善存取款、支付等基本金融服务。稳定农村信用社县域法人地位，提高治理水平和服务能力。开展农村信用社省联社改革试点，逐步淡出行政管理，强化服务职能。鼓励国有和股份制金融机构拓展“三农”业务。深化中国农业银行三农金融事业部改革，加大“三农”金融产品创新和重点领域信贷投入力度。发挥国家开发银行优势和作用，加强服务“三农”融资模式创新。强化中国农业发展银行政策性职能，加大中长期“三农”信贷投放力度。支持中国邮政储蓄银行建立三农金融事业部，打造专业化为农服务体系。创新村镇银行设立模式，扩大覆盖面。引导互联网金融、移动金融在农村规范发展。扩大在农民合作社内部开展信用合作试点的范围，健全风险防范化解机制，落实地方政府监管责任。开展农村金融综合改革试验，探索创新农村金融组织和服务。发展农村金融租赁业务。在风险可控前提下，稳妥有序推进农村承包土地的经营权和农民住房财产权抵押贷款试点。积极发展林权抵押贷款。创设农产品期货品种，开展农产品期权试点。支持涉农企业依托多层次资本市场融资，加大债券市场服务“三农”力度。全面推进农村信用体系建设。加快建立“三农”融资担保体系。完善中央与地方双层金融监管机制，切实防范农村金融风险。强化农村金融消费者风险教育和保护。完善“三农”贷款统计，突出农户贷款、新型农业经营主体贷款、扶贫贴息贷款等。
　　25．完善农业保险制度。把农业保险作为支持农业的重要手段，扩大农业保险覆盖面、增加保险品种、提高风险保障水平。积极开发适应新型农业经营主体需求的保险品种。探索开展重要农产品目标价格保险，以及收入保险、天气指数保险试点。支持地方发展特色优势农产品保险、渔业保险、设施农业保险。完善森林保险制度。探索建立农业补贴、涉农信贷、农产品期货和农业保险联动机制。积极探索农业保险保单质押贷款和农户信用保证保险。稳步扩大“保险＋期货”试点。鼓励和支持保险资金开展支农融资业务创新试点。进一步完善农业保险大灾风险分散机制。
　　26．深化农村集体产权制度改革。到2020年基本完成土地等农村集体资源性资产确权登记颁证、经营性资产折股量化到本集体经济组织成员，健全非经营性资产集体统一运营管理机制。稳定农村土地承包关系，落实集体所有权，稳定农户承包权，放活土地经营权，完善“三权分置”办法，明确农村土地承包关系长久不变的具体规定。继续扩大农村承包地确权登记颁证整省推进试点。依法推进土地经营权有序流转，鼓励和引导农户自愿互换承包地块实现连片耕种。研究制定稳定和完善农村基本经营制度的指导意见。加快推进房地一体的农村集体建设用地和宅基地使用权确权登记颁证，所需工作经费纳入地方财政预算。推进农村土地征收、集体经营性建设用地入市、宅基地制度改革试点。完善宅基地权益保障和取得方式，探索农民住房保障新机制。总结农村集体经营性建设用地入市改革试点经验，适当提高农民集体和个人分享的增值收益，抓紧出台土地增值收益调节金征管办法。完善和拓展城乡建设用地增减挂钩试点，将指标交易收益用于改善农民生产生活条件。探索将通过土地整治增加的耕地作为占补平衡补充耕地的指标，按照谁投入、谁受益的原则返还指标交易收益。研究国家重大工程建设补充耕地由国家统筹的具体办法。加快编制村级土地利用规划。探索将财政资金投入农业农村形成的经营性资产，通过股权量化到户，让集体组织成员长期分享资产收益。制定促进农村集体产权制度改革的税收优惠政策。开展扶持村级集体经济发展试点。深入推进供销合作社综合改革，提升为农服务能力。完善集体林权制度，引导林权规范有序流转，鼓励发展家庭林场、股份合作林场。完善草原承包经营制度。

　　六、加强和改善党对“三农”工作领导
　　加快农业现代化和农民奔小康，必须坚持党总揽全局、协调各方的领导核心作用，改进农村工作体制机制和方式方法，不断强化政治和组织保障。
　　27．提高党领导农村工作水平。坚持把解决好“三农”问题作为全党工作重中之重不动摇，以更大的决心、下更大的气力加快补齐农业农村这块全面小康的短板。不断健全党委统一领导、党政齐抓共管、党委农村工作综合部门统筹协调、各部门各负其责的农村工作领导体制和工作机制。注重选派熟悉“三农”工作的干部进省市县党委和政府领导班子。各级党委和政府要把握好“三农”战略地位、农业农村发展新特点，顺应农民新期盼，关心群众诉求，解决突出问题，提高做好“三农”工作本领。巩固和拓展党的群众路线教育实践活动和“三严三实”专题教育成果。进一步减少和下放涉农行政审批事项。加强“三农”前瞻性、全局性、储备性政策研究，健全决策咨询机制。扎实推进农村各项改革，鼓励和允许不同地方实行差别化探索。对批准开展的农村改革试点，要不断总结可复制、可推广的经验，推动相关政策出台和法律法规立改废释。深入推进农村改革试验区工作。全面提升农村经济社会发展调查统计水平，扎实做好第三次全国农业普查。加快建立全球农业数据调查分析系统。加强农村法治建设，完善农村产权保护、农业市场规范运行、农业支持保护、农业资源环境等方面的法律法规。
　　28．加强农村基层党组织建设。始终坚持农村基层党组织领导核心地位不动摇，充分发挥农村基层党组织的战斗堡垒作用和党员的先锋模范作用，不断夯实党在农村基层执政的组织基础。严格落实各级党委抓农村基层党建工作责任制，发挥县级党委“一线指挥部”作用，实现整乡推进、整县提升。建立市县乡党委书记抓农村基层党建问题清单、任务清单、责任清单，坚持开展市县乡党委书记抓基层党建述职评议考核。选优配强乡镇领导班子尤其是党委书记，切实加强乡镇党委思想、作风、能力建设。选好用好管好农村基层党组织带头人，从严加强农村党员队伍建设，持续整顿软弱涣散村党组织，认真抓好选派“第一书记”工作。创新完善基层党组织设置，确保党的组织和党的工作全面覆盖、有效覆盖。健全以财政投入为主的经费保障制度，落实村级组织运转经费和村干部报酬待遇。进一步加强和改进大学生村官工作。各级党委特别是县级党委要切实履行农村基层党风廉政建设的主体责任，纪委要履行好监督责任，将全面从严治党的要求落实到农村基层，对责任不落实和不履行监管职责的要严肃问责。着力转变基层干部作风，解决不作为、乱作为问题，加大对农民群众身边腐败问题的监督审查力度，重点查处土地征收、涉农资金、扶贫开发、“三资”管理等领域虚报冒领、截留私分、贪污挪用等侵犯农民群众权益的问题。加强农民负担监管工作。
　　29．创新和完善乡村治理机制。加强乡镇服务型政府建设。研究提出深化经济发达镇行政管理体制改革指导意见。依法开展村民自治实践，探索村党组织领导的村民自治有效实现形式。深化农村社区建设试点工作，完善多元共治的农村社区治理结构。在有实际需要的地方开展以村民小组或自然村为基本单元的村民自治试点。建立健全务实管用的村务监督委员会或其他形式的村务监督机构。发挥好村规民约在乡村治理中的积极作用。深入开展涉农信访突出问题专项治理。加强农村法律服务和法律援助。推进县乡村三级综治中心建设，完善农村治安防控体系。开展农村不良风气专项治理，整治农村黄赌毒、非法宗教活动等突出问题。依法打击扰乱农村生产生活秩序、危害农民生命财产安全的犯罪活动。
　　30．深化农村精神文明建设。深入开展中国特色社会主义和中国梦宣传教育，加强农村思想道德建设，大力培育和弘扬社会主义核心价值观，增强农民的国家意识、法治意识、社会责任意识，加强诚信教育，倡导契约精神、科学精神，提高农民文明素质和农村社会文明程度。深入开展文明村镇、“星级文明户”、“五好文明家庭”创建，培育文明乡风、优良家风、新乡贤文化。广泛宣传优秀基层干部、道德模范、身边好人等先进事迹。弘扬优秀传统文化，抓好移风易俗，树立健康文明新风尚。
　　让我们更加紧密地团结在以习近平同志为总书记的党中央周围，艰苦奋斗，真抓实干，攻坚克难，努力开创农业农村工作新局面，为夺取全面建成小康社会决胜阶段的伟大胜利作出更大贡献！

© 北大法宝：（[www.pkulaw.com](https://www.pkulaw.com/)) 专业提供法律信息、法学知识和法律软件领域各类解决方案。北大法宝为您提供丰富的参考资料，正式引用法规条文时请与标准文本核对。
欢迎查看所有[产品和服务](https://www.pkulaw.com/helps/2.html)。法宝快讯：[如何快速找到您需要的检索结果？](https://www.pkulaw.com/helps/70.html)[法宝](https://www.pkulaw.com/helps/69.html)V6有何新特色？

扫描二维码阅读原文

**原文链接：**<https://www.pkulaw.com/chl/89ecca84d3445c2fbdfb.html>
